# Distribution pattern following systemic mesenchymal stem cell injection depends on the age of the recipient and neuronal health

**DOI:** 10.1186/s13287-017-0533-2

**Published:** 2017-04-18

**Authors:** Claire Fabian, Yahaira Naaldijk, Christiane Leovsky, Adiv A. Johnson, Lukas Rudolph, Carsten Jaeger, Katrin Arnold, Alexandra Stolzing

**Affiliations:** 1 Interdisciplinary Centre for Bioinformatics (IZBI), University of Leipzig, Leipzig, Germany; 2 Fraunhofer Institute for Cell Therapy and Immunology (IZI), Leipzig, Germany; 3 Department of Ophthalmology, Mayo Clinic, Rochester, MN USA; 4 Paul Flechsig Institute for Brain Research, University of Leipzig, Leipzig, Germany; 5 Centre for Biological Engineering, Wolfson School, Loughborough University, Loughborough, UK

**Keywords:** Mesenchymal stem cells, Biodistribution, Systemic injection, Aging, Alzheimer’s disease

## Abstract

**Background:**

Mesenchymal stem cells (MSCs) show therapeutic efficacy in many different age-related degenerative diseases, including Alzheimer’s disease. Very little is currently known about whether or not aging impacts the transplantation efficiency of MSCs.

**Methods:**

In this study, we investigated the distribution of intravenously transplanted syngeneic MSCs derived from young and aged mice into young, aged, and transgenic APP/PS1 Alzheimer’s disease mice. MSCs from male donors were transplanted into female mice and their distribution pattern was monitored by PCR using Y-chromosome specific probes. Biodistribution of transplanted MSCs in the brains of APP/PS1 mice was additionally confirmed by immunofluorescence and confocal microscopy.

**Results:**

Four weeks after transplantation into young mice, young MSCs were found in the lung, axillary lymph nodes, blood, kidney, bone marrow, spleen, liver, heart, and brain cortex. In contrast, young MSCs that were transplanted into aged mice were only found in the brain cortex. In both young and aged mouse recipients, transplantation of aged MSCs showed biodistribution only in the blood and spleen. Although young transplanted MSCs only showed neuronal distribution in the brain cortex in young mice, they exhibited a wide neuronal distribution pattern in the brains of APP/PS1 mice and were found in the cortex, cerebellum, hippocampus, olfactory bulb, and brainstem. The immunofluorescent signal of both transplanted MSCs and resident microglia was robust in the brains of APP/PS1 mice. Monocyte chemoattractant-1 levels were lowest in the brain cortex of young mice and were significantly increased in APP/PS1 mice. Within the hippocampus, monocyte chemoattractant-1 levels were significantly higher in aged mice compared with younger and APP/PS1 mice.

**Conclusions:**

We demonstrate in vivo that MSC biodistribution post transplantation is detrimentally affected by aging and neuronal health. Aging of both the recipient and the donor MSCs used attenuates transplantation efficiency. Clinically, our data would suggest that aged MSCs should not be used for transplantation and that transplantation of MSCs into aged patients will be less efficacious.

**Electronic supplementary material:**

The online version of this article (doi:10.1186/s13287-017-0533-2) contains supplementary material, which is available to authorized users.

## Background

Mesenchymal stem cells (MSCs) are a heterogenic mix of progenitor and stem cells that can differentiate into various mesenchymal tissues, including cartilage, bone, and adipose tissue [1]. Transplantation of MSCs has been investigated for a variety of diseases and many of these approaches have already entered clinical trials [2]. MSCs are often administered systemically for clinical application and systemic delivery of MSCs has been performed for a variety of ailments, including osteogenesis imperfecta [3], bone defects [4], diabetes [5], myocardial infarctions [6], multiple sclerosis [7], and arthritis [8]. The underlying regenerative mechanism of action seems to be related to the immune-modulatory, anti-inflammatory, and anti-fibrotic activity of MSCs [9, 10].

The success of a given MSC transplantation therapy may depend on a variety of factors, such as whether or not they were pre-incubated with protective compounds [11, 12] or under specific environmental conditions [13]. Another key factor underlying MSC transplantation efficiency may be the ability of MSCs to localize, or migrate, into target tissues of interest [14]. Intravenously transplanted MSCs have been found in very low frequencies in different organs using various labelling and tracking methods, including fluorescently labelled cells, xenogeneic transplantation, and sex-linked chromosome detection tracked by PCR [15–19]. Twenty-four hours after intravenous transplantation, MSCs are mostly found in the lung, liver, kidney, skin, thymus, lymph node, and gut with 1–2.7% of transplanted cells homing to these different organs in young animals [20]. MSCs first accumulate in the lung 24–48 hours after transplantation but can be found later in the liver, kidney, spleen, and other organs, particularly those showing injury [21]. Multimodal MRI nanoparticles with enhanced near-infrared fluorescence have been used recently to perform in-vivo imaging of human adipose-derived stem cells in an Alzheimer’s disease mouse model [22]. Cells administered via tail-vein injection were observed in the tail, body, and brain of Alzheimer’s disease mice up to 10 days after transplantation (with the strongest signal at day 3), but not in the brains of wild-type (WT) controls [22]. Post-mortem examination of organs revealed weak fluorescent signals in WT brain tissue, suggesting that some cells are able to cross the blood–brain barrier (BBB) in young animals. However, the strongest signals were observed in the Alzheimer’s disease mouse brains, which the authors attribute to leakage of the BBB brought about as a result of neurological disease. In addition, human adipose-derived stem cells had transmigrated to the gastrointestinal tract, kidney, liver, and bladder of all injected mice [22].

MSCs mediate numerous therapeutic effects by promoting repair directly via differentiation into critical cell types or indirectly through the secretion of substances and the activation of endogenous mechanisms [23]. In order to mediate such beneficial effects, MSCs must first home or migrate to a specific site of injury or damage. MSC migration is therefore thought to play a paramount role in the remedial process [23]. Migration of MSCs is subject to extensive regulation [13, 23, 24] and existing evidence suggests that MSCs migrate to specific organs in a manner similar to leukocytes. They adhere to endothelial cells in the vascular system and transmigrate across the vascular endothelium [25] towards injured and inflamed tissues [26, 27]. MSCs home to areas of injury along a chemokine gradient and several chemokines have been identified which affect MSC migration, including stromal cell derived factor 1 alpha, CXC chemokine receptor type 4, transforming growth factor beta 1, interleukin 1 beta, and tumour necrosis factor alpha [24, 28, 29]. Many of these specific regulatory factors have been shown to also affect the expression of chemokines and selectins in MSCs [13, 30–32].

Although conflicting reports exist in the literature as to whether or not MSC migration is affected by aging [13, 33], aging is known to detrimentally affect MSC functionality [1]. More broadly, aging is known to exert negative effects on stem cells and progenitor cells [34–36]. Aging increases the susceptibility of MSCs to damaging agents like reactive oxygen species, disrupts cell population dynamics, diminishes therapeutic efficacy, and mediates other harmful effects [1, 37–39]. While numerous studies have uncovered aspects of MSC aging, it remains to be determined whether or not aging affects MSC transplantation efficiency. Moreover, further data are required to clarify whether or not MSC migration and engraftment is affected in vivo by aging. To better elucidate these questions, we assessed MSC biodistribution in vivo in various mouse organs following transplantation with young or old MSCs into young, old, or diseased animals.

## Methods

### Animals

C57Bl/6 mice as a source for bone marrow were obtained from the University of Leipzig or Charles River. The GFP transgenic mice were from the Paul Flechsig Institute for Brain Research, University of Leipzig, and the transgenic mice overexpressing human amyloid precursor protein (APP_KM670/671NL_) and presenilin-1 (PS1_L166P_) under Thy-1 promoter control were obtained from University of Leipzig (Prof. Bechmann).

### MSC preparation

After isolation, bone marrow from tibiae and femurae were harvested as described previously [40] and cultured in DMEM 1×, low glucose (Gibco) with GlutamaxI (Gibco), 10% fetal calf serum (Biochrome), and 1% penicillin/streptomycin (Gibco) for the isolation of adherent MSCs. Briefly, mouse bone marrow cells were obtained by centrifugation from tibiae and femurae and were cultured according to the method of Dobson et al. [41]. MSCs were isolated by the method of Sekiya et al. [42]. MSCs were passaged when they were at 70% confluency using trypsin (0.25% trypsin–EDTA; Gibco). Like we have done previously [43], fluorescence-activated cell sorting was used to confirm the identity of MSCs by assessing specific markers (e.g., CD11b^–^, CD45^–^, CD44^+^, CD90^+^). Similarly to earlier studies [1, 13, 43], mesodermal lineage differentiation was also performed to confirm successful MSC isolation and culturing.

### MSC transplantation

To set up a sex-mismatched transplantation, 1 × 10^6^ MSCs (passages 2–3) from male donors were transplanted into female recipients by tail-vein injection. MSCs were isolated either from young mice (2–3 months old) or from aged mice (12–13 months old). Recipient mice were either young (3 months old) or old (13–21 months old). Older APP/PS1 mice (12–15 months old) also received young MSCs. Twenty-eight days after transplantation, the mice were sacrificed and the organs isolated for analysis. Three to five mice were used for each experimental group. Three male mice were also transplanted with 1 × 10^6^ bone-marrow derived MSCs from female GFP-transgenic mice (3 months old) and were sacrificed for histological analysis 28 days after transplantation.

### Preparation of tissue

For genomic DNA (gDNA) isolation, mice were sacrificed 28 days after transplantation and transcardially perfused with 0.9% NaCl. Peripheral organs (lung liver, kidney, heart, lymph nodes, bone marrow, spleen) were removed, as well as the brain which was divided into five regions (hippocampus, cortex, cerebellum, brainstem, olfactory bulb). The tissue was mechanically homogenized in peqGOLD TriFast™ (PeqLab) and stored at –80 °C until further use.

For histology, mice were sacrificed after 28 days and perfused transcardially with 0.9% NaCl followed by 4% paraformaldehyde and 0.1% glutaraldehyde in 0.1 M phosphate buffer (pH 7.4). Brains were removed and immersion-fixed overnight in the same fixative at 4 °C. Brains were cryoprotected in 30% sucrose in 0.1 M phosphate buffer (pH 7.4) with 0.1% sodium azide, cut into 40 μm slices with a cryomicrotome in the frontal plane, and collected in 0.1 M phosphate buffer (pH 7.4) with 0.1% sodium azide.

### gDNA isolation

gDNA was isolated using peqGOLD TriFast™ (PeqLab) according to the manufacturer’s instructions. After isolation, gDNA was fractionated through an injection needle (size 2; Braun) using a 0.01–1 ml syringe (Braun). gDNA was then either further purified using a gDNA clean-up kit (Macherey-Nagel) or purified and enriched using a gDNA clean-up XS kit (Macherey-Nagel). All kits were used according to the manufacturer’s instructions.

### RNA isolation and quantitative RT–PCR

RNA was extracted from organs using peqGOLDTrifast™ reagent according to the manufacturer’s instructions (30-2040; PeqLab) and treated with DNaseI (EN0521; Life Technologies). This was followed by cDNA synthesis using SuperscriptIII reverse transcriptase (18080085; Life Technologies) and Oligo (dT)18-Primers (SO132; Thermo Scientific) at 50 °C for 1 hour. cDNA was used as PCR template in a 1:10 dilution and each sample run in triplicate. Quantitative PCR was performed using Express SYBR GreenER qPCR Supermix Universal (1178401 K; Life Technologies), additional 1× SybrGreen (S-7567; Life Technologies), and 0.2 μM primer each on the DNA engine Opticon2 (Biorad) (see Table 1) with the following cycle conditions: primary denaturation at 95 °C for 3 min, 35 cycles for 30 s at 95 °C, 30 s at 60 °C, and 30 s at 72 °C, followed by fluorescence measurement. Absolute quantification was performed for every single gene with three technical repeats per sample. Serial dilutions of plasmid controls with known molecule concentrations were used as positive control and to generate standard curves for the PCR. Expression of target genes was normalized using 36B4 (large ribosomal protein P0, RPLP0) as a reference gene.

**Table 1:** PCR primers

Name	Gene	Sense primer (5'–3')	Antisense primer (5'–3')	NCBI accession number
36B4	Ribosomal protein large P0, RPLP0	CCGTGTGAGGTCACTGTGCCAGCTC	GCCCAAAGCCTGGAAGAAGGAGGTC	NM_007475.5
MCP-1	Transcription regulatory protein MCP-1 (POU 1)	GCAGTAATCCTCACCAGCCCAACGC	GATCCCGTCCTCATCCAGACTTGG	L13763.1

### Cell-tracking PCR

Male gDNA was tracked by PCR using Y-chromosome specific primers (5′-CATGGAGAGCCACAAGCTAACCA-3′ and 5′-GTCCCAGCATGAGAAAGATTCTTCC-3′). Up to four copies of Y-chromosomes can be detected in the background of up to 50 ng gDNA. The PCR mix contained 0.2 μM primers, 0.2 mM dNTPs (Thermo Scientific), 2.8 mM MgCl_2_ (Life Technologies), 1× buffer without MgCl_2_ (Life Technologies), and 1 U platinum taq polymerase (Life Technologies). gDNA (5 μl) was added to the mix as a template. As a positive control, 5 μl gDNA isolated from male mouse brain tissue was used (concentration: 0.55 ng/ml in TE buffer, pH 8.0) instead of the sample gDNA. The single copy gene 36B4 (5′-CCGTGTGAGGTCACTGTGCCAGCTC-3′ and 5′-GCCCAAAGCCTGGAAGAAGGAGGTC-3′) was used as reference gene. PCR was performed in a thermocycler T professional (Biometra) with a primary 3-min denaturation step at 95 °C followed by 35 cycles: 30 s at 95 °C, 30 s at 60 °C, and 30 s at 72 °C, with a final 3-min elongation step at 72 °C. The PCR products were verified by agarose gel electrophoresis.

### Immunohistochemistry

Immunohistochemistry was performed with free-floating sections. Brain slices were washed once with PBS containing 0.05% Tween20 (PBS-T) (P2287-100ML; Sigma-Aldrich) and treated with 60% methanol (VWR Prolabo) for 1 hour. Sections were incubated in blocking solution consisting of PBS-T plus 2% bovine serum albumin (11930; Serva), 0.3% milk powder (T145; Roth), and 0.5% donkey normal serum (017-000-001; Jackson Immuno Research) for 30 min prior to incubation with the primary antibody. Immunofluorescence staining for microglia was performed with rabbit anti-Iba-1 (1:500; WAKO) in blocking solution overnight at 4 °C, followed by secondary antibody donkey-anti-rabbit Cy3 (1:250, 711-165-152; Dianova) for 1 hour. Finally, slices were washed three times and incubated for 10 min with DAPI (1:10,000; Sigma) at 4 °C and washed once. For immigrated GFP^+^ MSCs, no immunohistochemical enhancement was used. Slices were mounted on glass slides, dried, and coverslipped with entellan in toluol (108323; Merck).

### Microscopy and image processing

Tissue sections were examined with the Keyence BZ-9000 microscope (Keyence Corporation, USA). Fluorescence labelling was examined with a Zeiss confocal laser scanning microscope (LSM 510; Zeiss, Jena, Germany). For imaging of GFP (green fluorescence), an argon laser with 488-nm excitation was used and emission from Cy2 was recorded at 510 nm with a low-range band pass filter (505–550 nm). For secondary Cy3-labelling (red fluorescence), a helium–neon laser with 543-nm excitation was used and emission from Cy3 at 570 nm was detected applying a high-range band pass (560–615 nm). For DAPI labelling, a 405-nm diode laser was used. Photoshop CS2 (Adobe Systems, Mountain View, CA, USA) was used to process the images with minimal alterations to the brightness, sharpness, colour saturation, and contrast.

### Statistical analysis

Data are presented as the mean of the samples and standard deviation as the standard error of the mean (SEM). Statistical analysis was performed using SigmaPlot 11.0 software (Systat Software Inc.) using one-way ANOVA. *p* < 0.05 was considered statistically significant.

## Results

### Post-transplantation health and MSC phenotyping

None of the mice receiving MSCs died or showed any pathological changes post transplantation. All MSCs used for transplantation were at culture passages 2–3 and exhibited tri-lineage differentiation potential into adipocytes, chondrocytes, and osteocytes (Additional file 1: Figure S1). MSCs transplanted were positive for CD29, CD44, CD73, CD105, and CD106 and were negative for CD45 and CD11 (Additional files 2 and 3: Figure S2 and S3).

### Biodistribution of systemically transplanted young MSCs into young and old mice

**Fig. 1:**
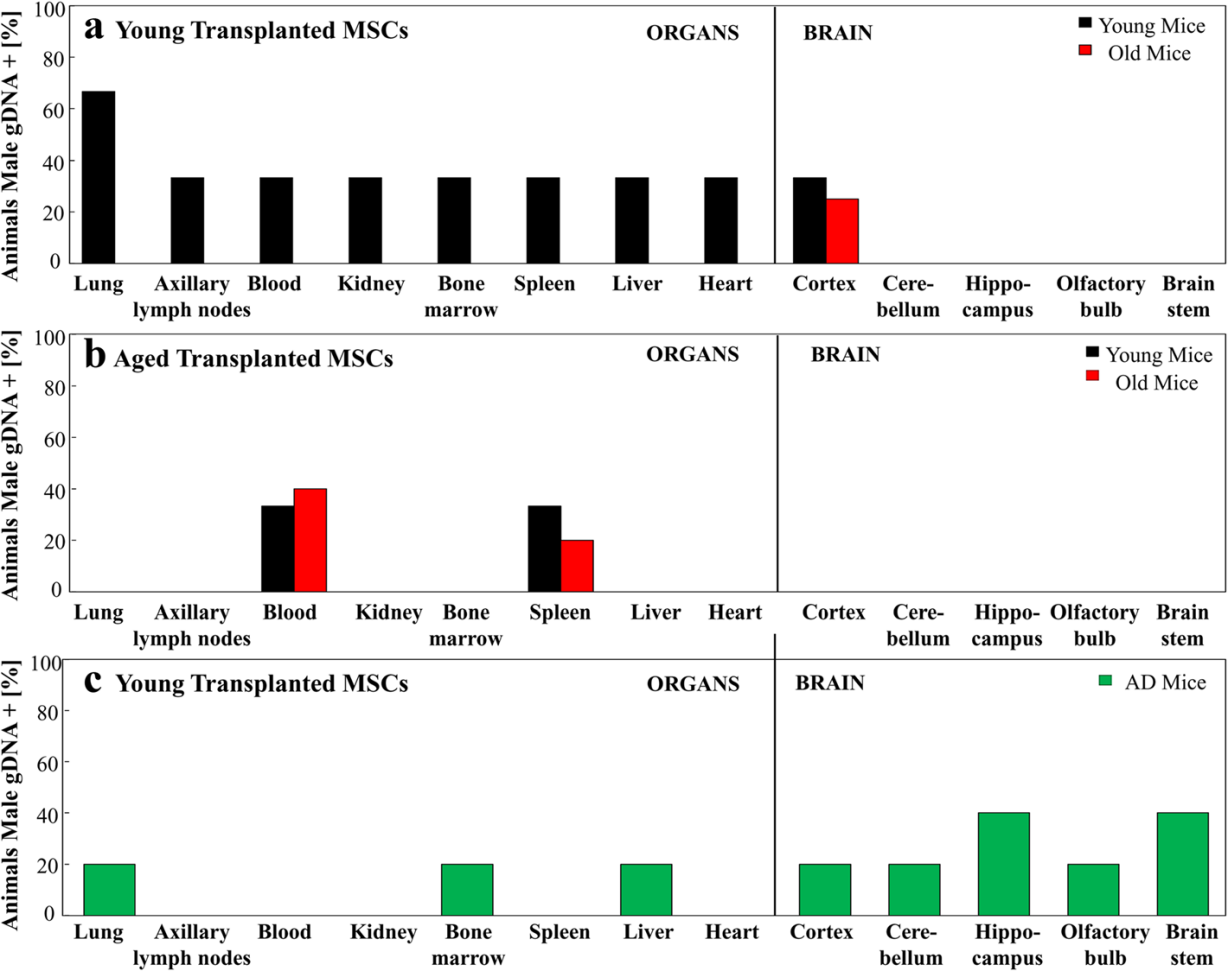
Biodistribution of young and old MSCs transplanted into young, old, and APP/PS1 mice. MSCs derived from young or old male mice were transplanted into young, old, and Alzheimer’s disease female mice via tail-vein injections. Percentage of male gDNA in organs was assessed and quantified 28 days after transplantation. (**a**) Young MSCs were intravenously transplanted into either young (*n* = 3) or old (*n* = 4) recipient mice. Young MSCs were found in the lungs, axillary lymph nodes, blood, kidney, bone marrow, spleen, liver, heart, and brain cortex of mice. In contrast, young MSCs were only found in the brain cortex of old mice. (**b**) Aged MSCs were transplanted into young (*n* = 3) and old (*n* = 5) mice. Biodistribution of aged MSCs was exclusive to the blood and spleen. (**c**) Young MSCs were injected into the tail veins of APP/PS1 Alzheimer’s disease (*AD*) mice (*n* = 5). Biodistribution was found in the cortex, cerebellum, hippocampus, olfactory bulb, brain stem, liver, bone marrow, and lung. *gDNA* genomic DNA, *MSC* mesenchymal stem cell

MSCs from young male donors were transplanted into young and old female recipients via tail-vein injection. As described in the Methods, Y-chromosome PCR analysis was then performed to assess the biodistribution of MSCs in isolated organs and tissues post mortem. Following a tail-vein injection, young transplanted MSCs were found in the lung, axillary lymph nodes, blood, kidney, bone marrow, spleen, liver, heart, and brain cortex of young mice (Fig. 1a). In contrast, male gDNA was only present in the brain cortex of old mice following transplantation with young MSCs (Fig. 1a).

### Biodistribution of systemically transplanted old MSCs into young and old mice

When aged MSCs were transplanted into young or aged mice, transplanted MSCs were only found in the blood and spleen (Fig. 1b). Although more MSCs were found in the spleen of young recipient mice, more MSCs were present in the blood of aged recipient mice (Fig. 1b). When young MSCs were transplanted into young and old mice, biodistribution in the brain was exclusive to the cortex (Fig. 1a). Conversely, aged MSCs were not found in any neuronal tissue post transplantation into either young or old mice (Fig. 1b). In comparison with young MSCs transplanted into young mice (Fig. 1a), aged MSCs transplanted into young mice showed a markedly decreased biodistribution (Fig. 1b).

### Biodistribution of systemically transplanted young MSCs into old APP/PS1 mice

In C57Bl/6 recipients, transplanted young MSCs showed neuronal distribution in the brain cortex (Fig. 1a) while transplanted old MSCs were not found in any brain tissues (Fig. 1b). In contrast, young MSCs which were transplanted into aged APP/PS1 mice were predominantly found in neuronal tissues. In addition to being distributed in the lung, bone marrow, and kidney, transplanted MSCs in these mice were found in all isolated brain parts, including the cortex, cerebellum, hippocampus, olfactory bulb, and brainstem (Fig. 1c). The strongest signals were found in the hippocampus and brainstem. APP/PS1 mice represent a mouse model of Alzheimer’s disease and, in this murine model of neuronal degeneration, MSCs transplanted via tail-vein injections homed preferentially into the brain.

**Fig. 2:**
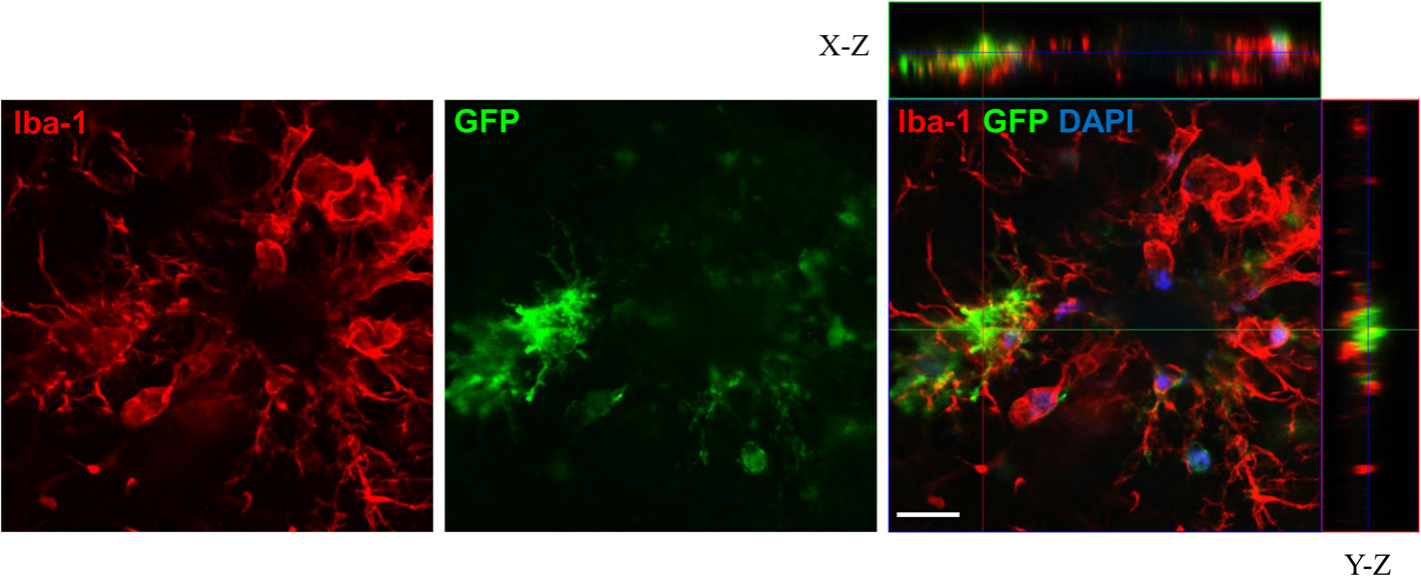
Migration of transplanted MSCs into the brain of APP/PS1 mice. Intravenously transplanted GFP ^+^ MSCs were able to migrate into the brain parenchyma. GFP^+^ MSCs (*green*, *centre panel*) were found in association with activated microglia (Iba-1; *red*, *left panel*), but only partly showing overlap with Iba-1 immunoreactivity (*right panel*). In the merged image (orthoview based on stack analysis), Iba-1 and GFP are shown in combination with nuclei (DAPI, *blue*) and, in addition to the X–Y image, orthogonal X–Z and Y–Z projections are shown. *Scale bar*: 20 μM (Colour figure online)

To further confirm that MSCs were indeed homing to the brain following a tail-vein injection, we employed immunofluorescence staining in addition to our Y-chromosome PCR analysis. GFP^+^ MSCs from young GFP-positive mice were transplanted into aged APP/PS1 mice via tail-vein injection. Immunofluorescent staining for nuclei (DAPI) and Iba-1, a marker of microglia [44], was then performed on frontal sections. In correspondence with the Y-chromosome PCR results, immigrated GFP^+^ MSCs were found in all brain regions, especially in the cortex and hippocampus. GFP^+^ MSCs appeared strongly attracted to inflammation sites and seemed to be integrated into the network of resident microglia (Fig. 2). A representative, low-magnification image is also included to highlight the reliability of this staining pattern (Additional file 4: Figure S4).

### Expression of monocyte chemoattractant protein-1 in young, aged, and APP/PS1 mouse brains

**Fig. 3:**
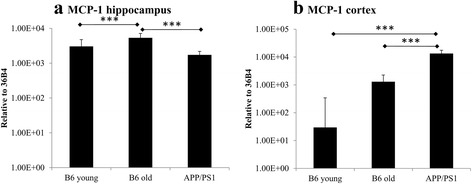
Monocyte chemoattractant protein-1 (*MCP-1*) levels in the hippocampus and cortex of young, aged, and APP/PS1 mice. Using PCR, the levels of the chemokine and MSC regulator MCP-1 were quantified in the hippocampus and cortex of young, aged, and APP/PS1 mice without cell treatment. **a** Compared with young and APP/PS1 mice, *MCP-1* levels were significantly increased in the hippocampus of old mice. **b** Within the brain cortex, young mice showed the lowest levels of *MCP-1* expression. Compared with both young and old mice, *MCP-1* levels were significantly increased in the brain cortex of APP/PS1 mice. ***p < 0.001

Monocyte chemoattractant protein-1 (MCP-1) is known to regulate the function and behaviour of MSCs [45, 46]. Using PCR, we measured *MCP-1* gene levels in the hippocampus and cortex of young, aged, and APP/PS1 mice (Fig. 3). We found that, in the hippocampus, MPC-1 levels were significantly higher (*p* < 0.001) in aged mice compared with *MCP-1* levels in young and APP/PS1 mice (Fig. 3a). In the cortex, *MCP-1* expression was the lowest in young mice (Fig. 3b). *MCP-1* expression was highest in APP/PS1 mice and was significantly greater (*p* < 0.001) than *MCP-1* expression in both young and aged mice (Fig. 3b).

### Biodistribution overview

**Fig. 4:**
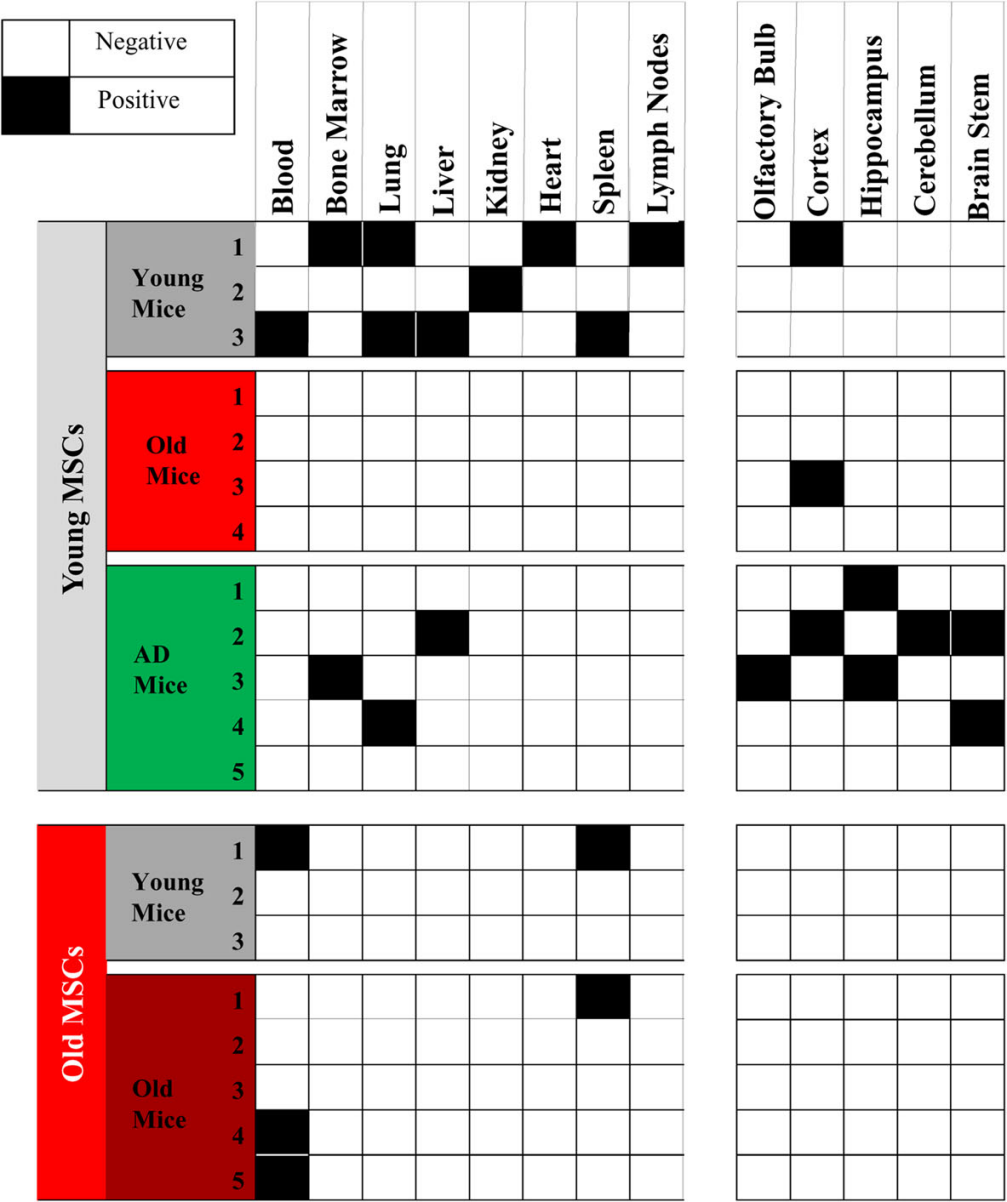
Biodistribution overview. Tabular biodistribution summary for each of the mice used for the transplantation experiments. *AD* Alzheimer’s disease, *MSC* mesenchymal stem cell

The results from the biodistribution experiments are summarized visually in Fig. 4. It is especially lucid that aging impacts transplantation efficiency when the data are presented in this tabular manner, because no young transplanted MSCs were found to home in any organ except the brain in any of the four aged mice tested (Fig. 4). Conversely, young MSCs were found in at least one organ in every young mouse recipient. Additionally, the overall engraftment was clearly lower in MSCs from aged donors compared with MSCs from young donors (Fig. 4). That MSCs migrated to various regions of the brain (young MSCs to the cortex of young and aged mice, and young MSCs to the cortex, cerebellum, hippocampus, olfactory bulb, and brain stem of APP/PS1 mice) is also very apparent (Fig. 4).

## Discussion

In our previous work, we demonstrated that aging detrimentally affects intravenously or intranasally transplanted in-vitro differentiated microglia derived from mouse bone marrow [47]. Specifically, we showed that transplanted microglia from young donors migrated to the brain in both young and old recipients while transplanted microglia from older donors failed to exhibit migration into the brain [47]. In the present work, we corroborate and expand upon this prior work [47] by presenting evidence that aging substantially hinders the transplantation efficiency of MSCs.

Although young MSCs migrated to nine organs when transplanted into young mice, young MSCs only migrated into a single organ in old mice. This result demonstrates that, even when young, robust MSCs are used for transplantation, the age of the recipient drastically affects transplantation efficiency and post-transplantation migration. Aspects of senescence in the elderly recipients must therefore be preventing the efficient migration or survival of MSCs. We recently showed that cytokines, growth factors, and O_2_ concentration affect MSC migration [13]. These and other age-dependent regulatory factors [35, 48] are probably anomalous in elderly mice and this dysregulation might be responsible for the restricted immigration of MSCs into the brain.

We further investigated how effectively intravenously transplanted aged MSCs would distribute in both young and old mice. Consistent with our findings regarding transplantation efficiency of young MSCs into aged mice, we found that aged MSCs revealed a dramatically reduced biodistribution in both young and aged recipients and were only found in two tissues—the blood and the spleen. Here, more young MSCs were found in the spleen of old mice and more old MSCs were found in the blood of old mice. We postulate that young MSCs were more capable of migrating into the spleen tissue while older MSCs were less able to leave the blood and home into this organ. Because this difference was not statistically significant, more research is required to determine whether this disparity is invariable.

These data indicate that, in addition to the age of the recipient host affecting MSC biodistribution, the age of the used MSCs themselves affects distribution into various tissues. Aging has been reported previously to detrimentally affect the ability of MSCs to mediate wound healing and vascular support [37]. Moreover, work by Bustos et al. [33] suggests that aging may impair the migratory and anti-inflammatory abilities of MSCs. Our data substantiate these and other claims that aging detrimentally affects MSC functionally. We add novel in-vivo data showing that the deleterious effects of aging significantly attenuate MSC biodistribution post transplantation.

Unlike aged C57Bl/6 mice, aged APP/PS1 mice showed a broad biodistribution following transplantation with young MSCs, with a preferential attraction towards the brain. Young MSCs were found in each of the examined brain areas (cortex, cerebellum, hippocampus, olfactory bulb, brainstem) following transplantation. Because some controversy still exists on whether or not MSCs can cross the BBB [49], we verified biodistribution in the brain by both Y-chromosome PCR and immunofluorescence detection of GFP^+^ MSCs. Our mice were not irradiated and not immune suppressed, which confirms that MSCs can cross the BBB in untreated, young, and aged mice. Concerning the APP/PS1 mice, the BBB is known to be affected in neurodegenerative disorders like Alzheimer’s disease. BBB breakdown due to disrupted tight junctions, altered transport processes, and inflammatory effects have been reported to underlie neurodegenerative diseases [50]. These factors would make it notably easier for MSCs to infiltrate the brain.

The preferential biodistribution in the brain of recipient APP/PS1 mice compared with recipient young and aged C57Bl/6 mice might also be in part due to differences in MCP-1 levels. In the brain cortex, APP/PS1 mice showed significantly higher *MCP-1* levels compared with old and young mice. Because MCP-1 is known to stimulate MSC migration [51], this increased *MCP-1* signal in the brain cortex of APP/PS1 mice might be a factor that attracts more MSCs into the brain. A confounding factor that does not support this hypothesis is that, although *MCP-1* levels were also increased in older mice compared with young mice, older mice recipients showed a significantly reduced biodistribution. It is important to note, however, that this difference in *MCP-1* levels between older and younger mice was not statistically significant.

Another paradoxical factor is that, in the mouse hippocampus, *MCP-1* levels were lowest in APP/PS1 mice and highest in aged mice. Aged mice exhibited significantly higher *MCP-1* levels than both young and APP/PS1 mice. One possible explanation for this is that amyloid plaques in this Alzheimer’s disease mouse model are more abundant in the cortex than in the hippocampus [52], which could explain why *MCP-1* levels were significantly increased in the brain cortex but not in the hippocampus of APP/PS1 mice. Amyloid-β can activate microglia, which in turn has been reported to increase MCP-1 production in rodents and humans [53]. Another possibility is that MCP-1 levels do not discernibly affect biodistribution and that the primary factor increasing neuronal distribution in APP/PS1 is disruption of the BBB due to neurodegeneration. Follow-up research is required to understand the detailed mechanisms underlying the observed differences in biodistribution between young, old, and APP/PS1 mice.

## Conclusions

In sum, we demonstrate in vivo that MSC biodistribution post transplantation is detrimentally affected by aging and neuronal health. Aging of both the recipient and the donor MSCs used attenuates transplantation efficiency. Clinically, our data would suggest that aged MSCs should not be used for transplantation and that transplantation of MSCs into aged patients will be less efficacious. Further studies are warranted to identify novel therapeutic strategies to improve biodistribution of MSCs in aged hosts. Additional studies are also warranted to see whether aging affects post-transplantation biodistribution in other disease models and to determine whether young and old MSCs preferentially home to organs of interest in aged versus young animals.

## Additional files

Additional file 1: Figure S1. showing mesodermal lineage differentiation of bone-marrow-derived MSCs. Bone-marrow-derived MSCs were differentiated in vitro under adipogenic (**a**), osteogenic (**b**), or chondrogenic (**c**) conditions. Verification of the differentiation was done by qualitative analysis: adipogenesis shown by staining lipid vesicles with Oil red-O (**a**), osteogenesis shown by staining alkaline phosphatase with Fast Red (**b**), and chondrogenesis shown by staining sulphated proteoglycans typical for extracellular matrix composition with Alcian Blue under acidic conditions.

Additional file 2: Figure S2. showing cell marker panels of Sca1, CD73, CD105, CD29, and CD45 in bone-marrow derived MSCs. A cell marker panel was performed via FACS on Bl6, bone-marrow-derived MSCs at passage 3. P2-Q4 represents the negative quadrant in the bottom left (*purple*). P2-Q1 represents the positive quadrant in the upper left (*green*). (TIF 1142 kb)

Additional file 3: Figure S3. showing cell marker panels of CD11b, CD106, and CD44 in bone-marrow-derived MSCs. A cell marker panel was performed via FACS on Bl6, bone-marrow-derived MSCs at passage 3. P2-Q4 represents the negative quadrant in the bottom left (*purple*). P2-Q1 represents the positive quadrant in the upper left (*green*). Bar graph (*bottom right*) and textual table (*bottom left*) included to summarize the overall results of the FACS experiments. (TIF 831 kb)

Additional file 4: Figure S4. showing a low-magnification image of transplanted MSCs in the brain of APP/PS1 mice. Representative low-magnification image showing the migration of transplanted GFP-positive MSCs (*green*) into the brains of APP/PS1 mice. GFP-expressing MSCs were found in association with activated microglia (Iba-1, *red*). Sections were stained with DAPI to highlight nuclei as a positional marker. *Scale bar*: 50 μM. (TIF 4445 kb)

## Abbreviations

BBB: Blood–brain barrier
MCP-1: Monocyte chemoattractant protein-1
MSC: Mesenchymal stem cell
WT: Wild type
