# Supplementary figures and images for: Distribution pattern following systemic mesenchymal stem cell injection depends on the age of the recipient and neuronal health
Source: Stem Cell Res Ther. 2017 Apr 18;8:85. doi: 10.1186/s13287-017-0533-2 (PMC5395862; doi:10.1186/s13287-017-0533-2)

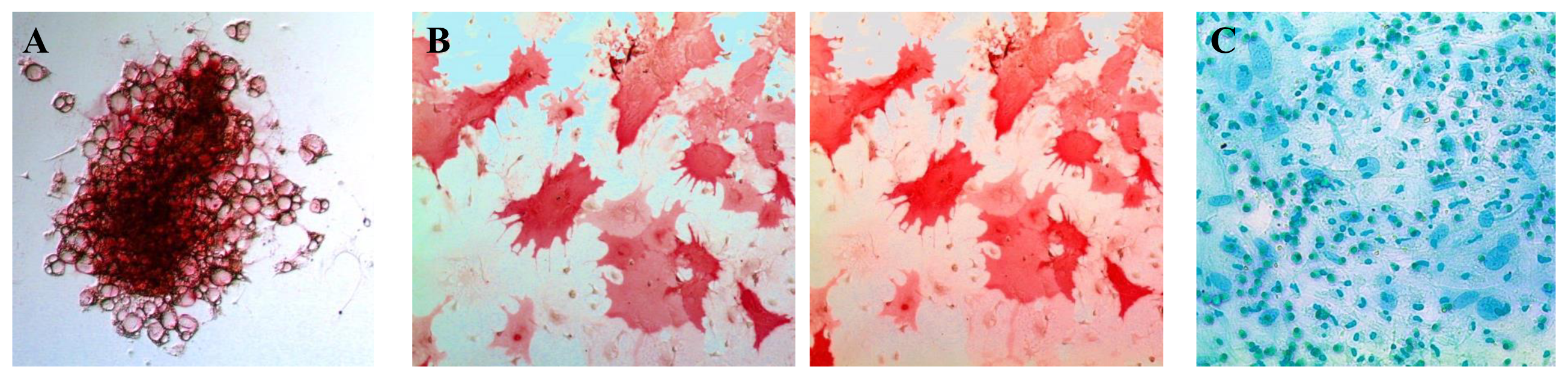

Supplement: Supplementary file 1 — showing mesodermal lineage differentiation of bone-marrow-derived MSCs. Bone-marrow-derived MSCs were differentiated in vitro under adipogenic (a), osteogenic (b), or chondrogenic (c) conditions. Verification of the differentiation was done by qualitative analysis: adipogenesis shown by staining lipid vesicles with Oil red-O (a), osteogenesis shown by staining alkaline phosphatase with Fast Red (b), and chondrogenesis shown by staining sulphated proteoglycans typical for extracellular matrix composition with Alcian Blue under acidic conditions. [file 13287_2017_533_MOESM1_ESM.tif]

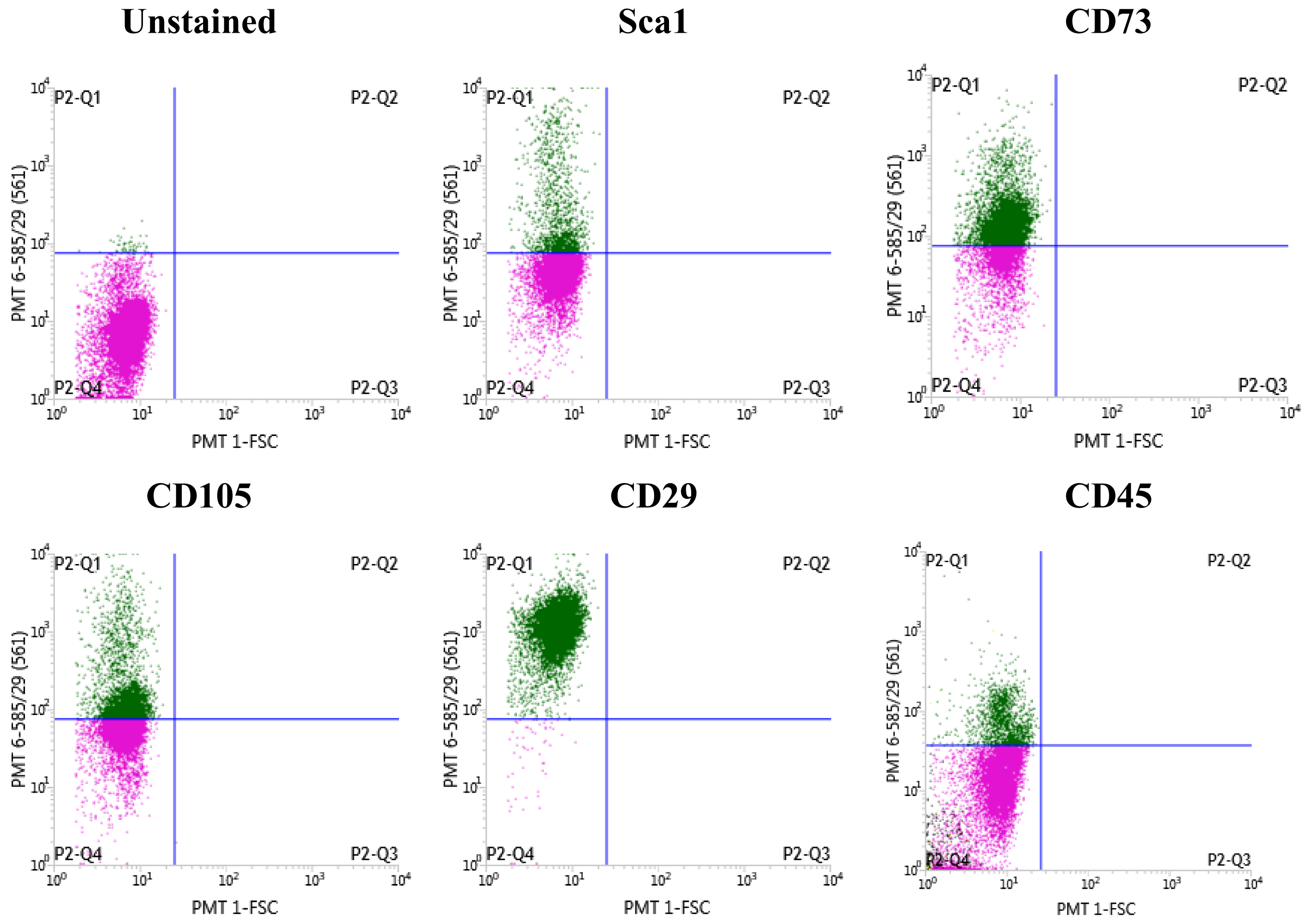

Supplement: Supplementary file 2 — showing cell marker panels of Sca1, CD73, CD105, CD29, and CD45 in bone-marrow derived MSCs. A cell marker panel was performed via FACS on Bl6, bone-marrow-derived MSCs at passage 3. P2-Q4 represents the negative quadrant in the bottom left (purple). P2-Q1 represents the positive quadrant in the upper left (green). (TIF 1142 kb) [file 13287_2017_533_MOESM2_ESM.tif]

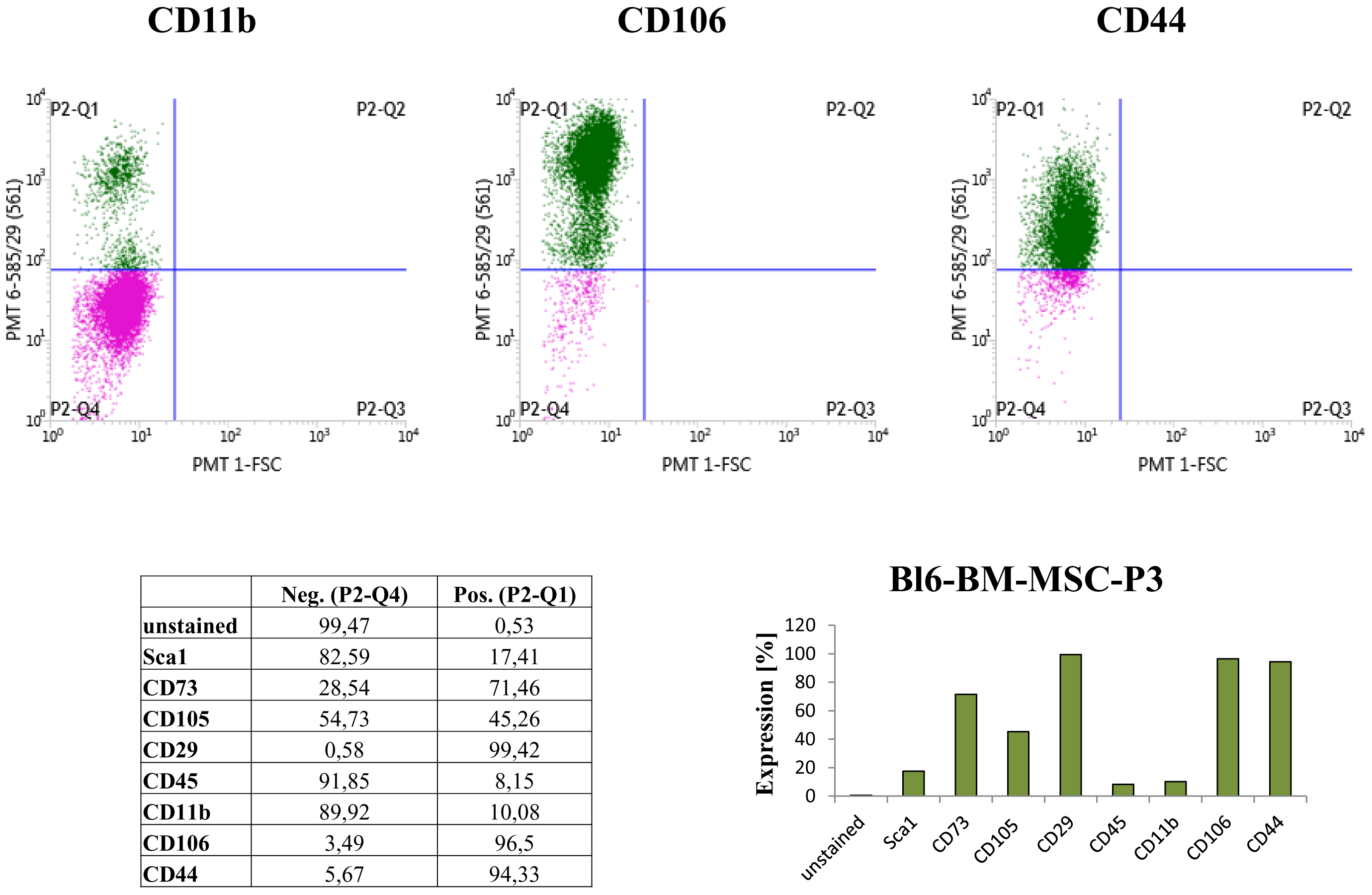

Supplement: Supplementary file 3 — showing cell marker panels of CD11b, CD106, and CD44 in bone-marrow-derived MSCs. A cell marker panel was performed via FACS on Bl6, bone-marrow-derived MSCs at passage 3. P2-Q4 represents the negative quadrant in the bottom left (purple). P2-Q1 represents the positive quadrant in the upper left (green). Bar graph (bottom right) and textual table (bottom left) included to summarize the overall results of the FACS experiments. (TIF 831 kb) [file 13287_2017_533_MOESM3_ESM.tif]

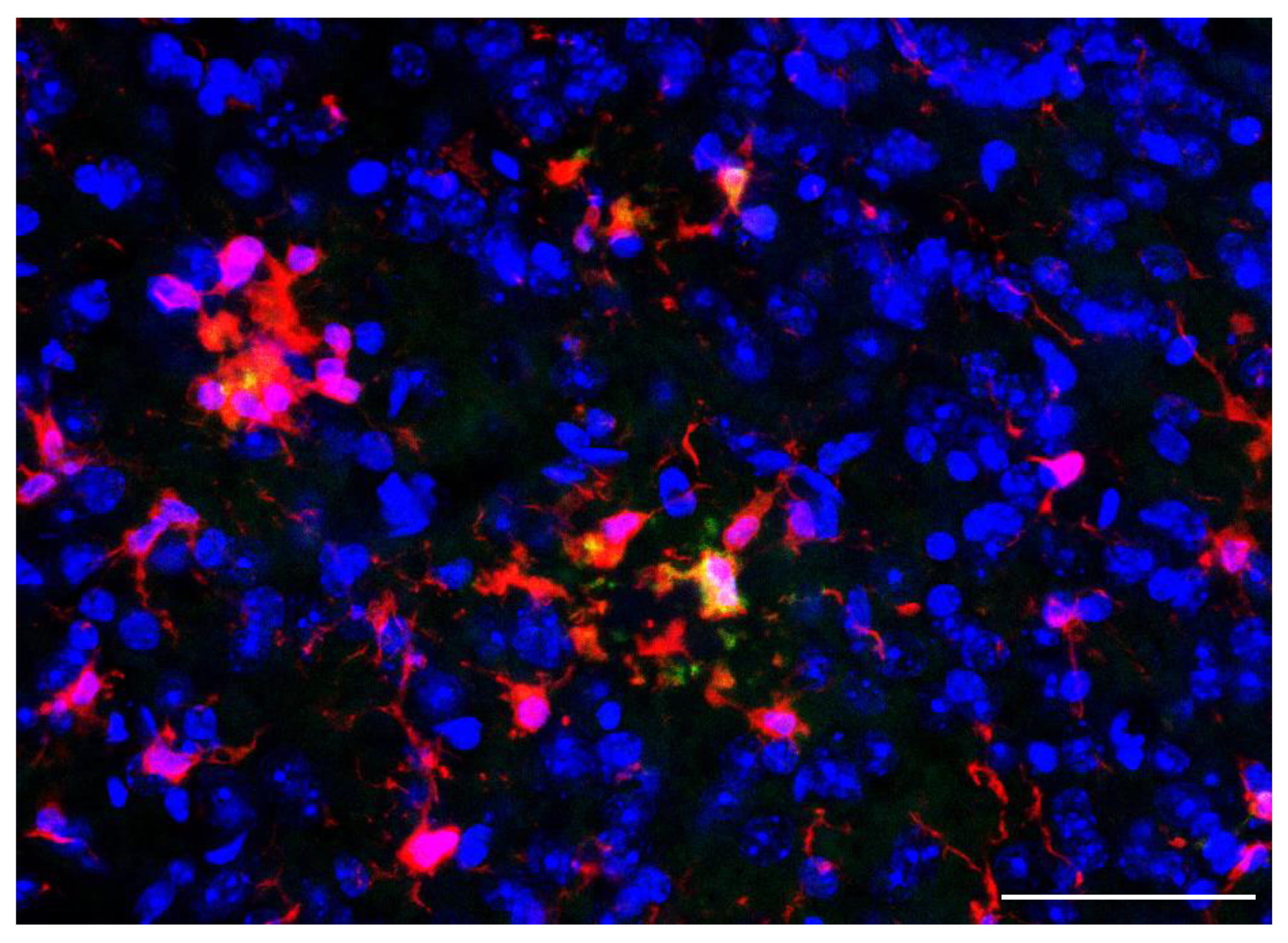

Supplement: Supplementary file 4 — showing a low-magnification image of transplanted MSCs in the brain of APP/PS1 mice. Representative low-magnification image showing the migration of transplanted GFP-positive MSCs (green) into the brains of APP/PS1 mice. GFP-expressing MSCs were found in association with activated microglia (Iba-1, red). Sections were stained with DAPI to highlight nuclei as a positional marker. Scale bar: 50 μM. (TIF 4445 kb) [file 13287_2017_533_MOESM4_ESM.tif]
